# Unhealthy Lifestyle Associated with Higher Intake of Sugar-Sweetened Beverages among Malaysian School-Aged Adolescents

**DOI:** 10.3390/ijerph16152785

**Published:** 2019-08-04

**Authors:** Wan Ying Gan, Siti Fathiah Mohamed, Leh Shii Law

**Affiliations:** Department of Nutrition and Dietetics, Faculty of Medicine and Health Sciences, Universiti Putra Malaysia, Serdang 43400, Malaysia

**Keywords:** Sugar-sweetened beverages (SSBs), obesity, physical activity, screen time, sleep quality, fast food, Malaysian adolescents

## Abstract

High consumption of sugar-sweetened beverages (SSBs) among adolescents has turned into a global concern due to its negative impact on health. This cross-sectional study determined the amount of SSB consumption among adolescents and its associated factors. A total of 421 adolescents aged 13.3 ± 1.3 years (41.8% males, 58.2% females) completed a self-administered questionnaire on sociodemographic characteristics, physical activity, screen-viewing behavior, sleep quality, frequency of eating at fast food restaurants, home food availability, peer social pressure, parenting practice, and SSB consumption. Weight and height were measured. Results showed that the mean daily consumption of SSBs among adolescents was 1038.15 ± 725.55 mL. The most commonly consumed SSB was malted drink, while the least commonly consumed SSB was instant coffee. The multiple linear regression results revealed that younger age (*β* = −0.204, *p* < 0.001), higher physical activity (*β* = 0.125, *p* = 0.022), higher screen time (*β* = 0.147, *p* = 0.007), poorer sleep quality (*β* = 0.228, *p* < 0.001), and unhealthy home food availability (*β* = 0.118, *p* = 0.032) were associated with a higher SSB intake. Therefore, promoting a healthy lifestyle may help to reduce the excessive consumption of SSBs among adolescents.

## 1. Introduction

Sugar-sweetened beverages (SSBs) are defined as any beverage that has added sugar or caloric sweetener such as “sucrose (50% glucose, 50% fructose), high-fructose corn syrup (HFCS; most often 45% glucose and 55% fructose), or fruit juice concentrates by manufacturers, establishments, or individuals and usually contain >25 kcal per 8 fluid ounces” [1]. Some instances of SSBs are calorie-containing carbonated drinks, sweetened milk, sweetened teas and coffees, sport drinks, energy drinks (energy juices and energy sodas), fruit-flavored drinks (fruit-flavored, non-carbonated, and non-alcoholic fruit drinks), sweetened fruit juices (nectars and mixed juices, juices with added caloric sweeteners, and non-dairy-based fruit smoothies), vitamin water drinks, sodas, and beverages with added sugar [1,2,3,4].

Sugar-sweetened beverages contribute to calories in diet, wherein consumption in large amounts leads to poor diet quality and obesity due to the added sugar content with little or nil nutritional value while being high in calories [1,2]. Apart from obesity, a high consumption of SSBs has adverse effects on children’s health, such as increased likelihood of dental caries, insulin resistance risk, and caffeine-related effects [5]. A cross-national analysis involving 75 nations carried out by Basu et al. [6] revealed that an increment in soft drink consumption from 9.5 to 11.4 gallons per person per year from 1997 to 2010 had been linked with the uprising overweight, obesity and diabetes issues at the global scale. Current evidence also suggests that a high consumption of SSBs, particularly the rise in dietary fructose consumption, has contributed to various health outcomes, including weight gain, high blood pressure, insulin resistance, diabetes, and liver disorders such as non-alcoholic steatohepatitis and non-alcoholic fatty liver disease [7]. A meta-analysis showed that every additional one serving per day of SSB intake increased the risk for incident hypertension (RR = 1.08, 95% CI = 1.04, 1.12) and cardiovascular disease (RR = 1.17, 95% CI = 1.10, 1.24) [8]. 

Adolescents seem to be the major consumers of SSBs, in which SSBs have become the major contributor of energy intake in their diet [4]. The Internet-based Family Life, Activity, Sun, Health, and Eating (FLASHE) study reported that 67.4% of adolescents (12–17 years old) in the United States consumed SSBs on a daily basis, 33.9% took between one and less than two SSBs, and 33.5% consumed two or more SSBs daily [9]. Likewise, the Ontario Student Drug Use and Health Survey revealed that 81.4 and 12.0% of middle- and high-school students (11–20 years old) consumed at least one SSB and energy drink during the preceding week, respectively [10]. In Malaysia, the National Health and Morbidity Survey (NHMS) 2011 discovered that 15.1% of adolescents consumed two or three cups of drinks with sweetened condensed milk per day [11]. The Global School-based Student Health Survey (GSHS) in 2012 found that 29.3% of Malaysian adolescents aged between 13 and 17 consumed carbonated soft drinks one or more times per day [12]. In terms of the amount of SSBs consumed, the Western Australia Pregnancy Cohort (Raine) Study reported that the consumption of SSBs was 335 g/day or 1.3 serving/day among adolescents aged between 14 and 17 [13]. In the Netherlands, the average SSB intake among children aged between 6 and 13 was 900 mL/day [14]. These findings suggest that the constant SSB intake poses a problem among adolescents globally and this uprising scenario warrants further investigation.

A number of prior studies have explored the factors associated with SSB consumption among adolescents, such as physical activity level [4,15,16], screen viewing [4,17,18], sleep quality [18], fast food intake [4,16], availability of SSBs at home [14,19,20], parenting practices [14,19], peer influence [19,20], and obesity [21]. Nevertheless, these reported findings appear to be inconsistent. Therefore, more studies are needed to correlate these identified factors with SSB consumption. Furthermore, only a handful of studies have looked into factors associated with SSB consumption among adolescents in Malaysia. As such, this study assessed the associations of lifestyle factors (physical activity level, screen-viewing behavior, sleep quality, and frequency of eating a meal or snack at fast food restaurant), socio-environmental factors (home food availability, peer social influence, and parenting practice), and body weight status with consumption of SSBs among adolescents. Extensive understanding of the factors related to SSB intake is an integral issue that can be of assistance in devising an effective nutrition-related intervention program to promote healthy lifestyle and eating behaviors among adolescents.

## 2. Materials and Methods

### 2.1. Participants

The probability proportional to size (PPS) sampling technique had been employed as the sampling method in this study. A list of 30 government public schools located within the Gombak district in Selangor state was obtained from the Malaysian Ministry of Education. Based on the PPS, a secondary school was randomly selected. Adolescents who were physically disabled and experienced learning disabilities or developmental delays were excluded from this study. Out of 604 eligible adolescents aged between 12 and 16, 501 agreed to participate in this study. A total of 421 adolescents were retained in the final analysis, since a number of adolescents failed to complete the questionnaires.

Ethics approval was obtained from the Ethics Committee for Research Involving Human Subjects, Universiti Putra Malaysia (Reference No.: JKEUPM-2017-172). Permission to carry out the study was granted from the Malaysian Ministry of Education and the Selangor Department of Education. Written informed consent forms were collected from each participant and their guardians. 

### 2.2. Measures

A set of Malay language self-administered questionnaires was completed by the participants to retrieve information pertaining to their sociodemographic background, lifestyle factors (physical activity, screen-viewing behavior, sleep quality, and frequency of eating a meal or snack at a fast food restaurant), socio-environmental factors (home food availability of SSB, peer social pressure and parenting practice), and consumption of SSBs. Additionally, the height and weight of the participants were measured by the researcher. 

#### 2.2.1. Sociodemographic Background

Self-reported information, such as age, sex, ethnicity, parent’s education level, monthly household income, and daily pocket money, were obtained from the participants.

#### 2.2.2. Intake of Sugar-Sweetened Beverages

The participants were required to report the frequency of SSB consumption on a weekly basis (for the past one week) and the amount of SSB intake in daily basis. The SSBs included fruit drinks, regular carbonated flavored drinks, isotonic drinks, soft drink bases, syrups, botanical beverage mixes, soy bean milk/drinks, cow’s milk, cultured milk, sweetened tea, instant coffee, and malted drinks, in accordance to Food Regulation 1985 [22] and MANS 2014 [23]. The amount of SSB consumption per day was based on household measurement (1 glass = 250 mL, 1 can = 325 mL, and 1 bottle = 500 mL). The amount of SSB consumption per day was determined by using the formula suggested by Tak et al. [24]:

Volume (mL/day) = (No. of days × total amount of beverages consumed)/7 days

#### 2.2.3. Physical Activity

The Physical Activity Questionnaire for Older Children (PAQ-C) [25] was used to assess physical activity level amongst the participants in the past seven days. The PAQ-C consisted of nine items. Item 1 was on spare time activities, while items 2 to 8 were regarding physical activity level during physical education class, recess, lunch, right after school, evening, and weekends. Lastly, item 9 was about the frequency of physical activities performed daily. The final PAQ-C activity summary score was obtained from the mean value of the total 9 items. A higher score in the PAQ-C indicated a higher physical activity level. Physical activity level was categorized into ‘low’ (1–2.33), ‘moderate’ (2.34–3.66), and ‘high’ (3.67–5.00) [26]. The Malay version of the PAQ-C applied in this study has been reported to be valid and reliable among Malaysian students aged between 10 and 17 [27].

#### 2.2.4. Screen-Viewing Behavior

Screen time was assessed by asking as to the frequency of watching television (including videos) and playing on smartphones or computer/video games during weekdays and weekends [18]. The total screen time was calculated by using the following formula:

Total screen time per day (hours) = 5/7 × (reported hours of PC + TV + smartphone time on weekdays) + 2/7 × (reported hours of PC + TV + smartphone time on weekend days)

#### 2.2.5. Sleep Quality

The 19-item Pittsburgh Sleep Quality Index (PSQI) [28] was used in this study to measure sleep quality. The PSQI consisted of seven components, namely, subjective sleep quality, sleep latency, sleep duration, habitual sleep efficiency, sleep disturbances, use of sleeping medication, and daytime dysfunction. The score for each component ranged between 0 and 3. The summation score, or better known as the Global PSQI Score, ranged from 0 to 21. A higher score indicated poorer sleep quality, while a global score of >5 was considered as “poor sleepers” and ≤5 reflected “good sleepers”. The validated Malay version of the PSQI [29] was employed in this study. The Cronbach’s alpha coefficient for this study was 0.634, indicating acceptable internal consistency reliability.

#### 2.2.6. Visiting Fast Food Restaurants

The frequency of eating a meal or snacking at fast food restaurants was assessed by using a single question derived from the GSHS Malaysia [12]: “During the past 7 days, on how many days did you eat food from a fast food restaurant, such as McDonalds, KFC and Pizza Hut?”. The response ranged from 0 to 7 days a week.

#### 2.2.7. Home Food Availability

The three subscales in the Project EAT survey [30], namely, healthy home food availability (5 items), unhealthy home food availability (4 items), as well as fruits and vegetables availability (5 items), were applied in this study. A 4-point Likert scale, from 1 (never available) to 4 (always available), was used. The score was calculated by summing the scores for all the items under each subscale. A higher score for a subscale indicated higher food availability for the food items represented by that particular subscale. In this study, the Cronbach’s alpha coefficient for healthy home food availability subscale was 0.707, unhealthy home food availability subscale was 0.771, while fruits and vegetables availability subscale was 0.739, thus signifying good internal consistency reliability.

#### 2.2.8. Peer Social Influence

Peer social influence was evaluated by using two questions derived from Luszczynska et al. [20]: ‘My friend discourages from eating snacks or drinking fizzy drink, lemonade or energy drink’ and ‘My friend disapproves of my eating snack or drinking fizzy drink’. The responses applied a 5-point Likert scale, from 1 (strongly disagree) to 5 (strongly agree). The mean score was calculated from the two questions. A higher score indicated higher peer social influence on the consumption of SSBs. The Spearman–Brown coefficient for this scale was 0.741, indicating good internal consistency reliability.

#### 2.2.9. Parenting Practices

Perceived restrictive food parenting practice was measured by using a 9-item questionnaire [31]. Parenting practice towards dietary behavior on SSB consumption for fathers and mothers was examined by employing two sets of similar questions (4 items). The final item was regarding the availability of SSBs at home. A 5-point Likert scale was used, from strongly disagree (1) to strongly agree (5). Additionally, a reversed score was applied for items 5, 6, and 9. A summation score was calculated by summing the score for all the items. A higher score indicated greater restrictive food parenting practice. In this study, the Cronbach’s alpha coefficient for restrictive food parenting practice was 0.601, signifying acceptable internal consistency reliability.

### 2.3. Anthropometric Measurements

The height of the participants was measured by using a SECA 206 Body Meter (SECA, Hamburg, Germany), while the weight was measured by using a TANITA Digital Weight Scale HD-319 (TANITA Corporation, Arlington Heights, IL, USA). All the anthropometric measurements were taken twice to compute their mean values. Body mass index-for-age z-scores (BAZ) of the participants were calculated by using the WHO AnthroPlus Version 1.0.4 software (WHO, Geneva, Switzerland). The classification of BAZ adhered to the WHO Growth Reference 2007 [32].

### 2.4. Statistical Analysis

IBM SPSS Statistics 24 (IBM SPSS Statistic, Inc., Chicago, IL, USA) was applied to perform all statistical analyses. Numerical descriptive data were presented in means, standard deviations, medians, and interquartile ranges, while categorical descriptive data were presented in count (n) and proportion (%). Simple linear regression was applied to determine the factors associated with the consumption of SSBs. All variables with *p* < 0.25 in the simple linear regression model were included in the multiple linear regression model. The significance level was set at *p* < 0.05. 

## 3. Results

Table 1 shows the characteristics of the participants. A total of 421 participants with a mean age of 13.3 ± 1.3 years participated in this study. Almost all the participants were Malays (97.9%). The mean value for self-reported daily pocket money was MYR 5.29 ± 2.44 (≈ 1.30 USD). A series of unhealthy lifestyle behaviors were reported by the participants. Two-thirds of the participants (68.1%) had low physical activity level, while one-third of them (35.9%) were poor sleepers. The total screen time of the participants was 3.67 ± 1.87 h daily. A total of 79.0% of the participants consumed fast food in the previous week. The prevalence of overweight, obesity, and severe obesity were 18.1, 15.7, and 4.0%, respectively. 

The mean total amount for daily consumption of SSBs was 1038.15 ± 725.55 mL (Table 2), approximately equal to 4 servings of intake (1 serving = 250 mL) in a day. The most popular SSB consumed by the participants was malted drinks, followed by milk and tea, whilst the least popular SSBs were instant coffee and botanical beverages. In fact, malted drinks (14.7%) and cow’s milk (11.9%) were consumed on a daily basis (Table 3). Nearly one-third of the participants (31.4%) did not consume fruit juice/fruit drink in a week.

The results obtained from multiple linear regression revealed that younger age (*β* = −0.204, *p* < 0.001), higher physical activity (*β* = 0.125, *p* = 0.022), higher screen time (*β* = 0.147, *p* = 0.007), poorer sleep quality (*β* = 0.228, *p* < 0.001), and unhealthy home food availability (*β* = 0.118, *p* = 0.032) were associated with higher SSB intake (Table 4).

## 4. Discussion

A high consumption of SSBs was observed in this study. The finding appears to exceed that reported in a local study that involved secondary school students in Kuala Lumpur, wherein the mean consumption was reported to be 177.5 mL per day [33]. A study in the Netherlands among children aged between 6 and 13 revealed that the consumption of SSBs was 900 mL per day [14], which is slightly lower than that retrieved in the present study. Nevertheless, it is essential to acknowledge that a comparison between studies has to be made cautiously as the variances that exist between the studies may be attributable to the varying definition, measurement, and classification of SSBs. For instance, SSBs in Loh et al. [33] referred to carbonated drinks, sugar-sweetened fruit drinks, non-dairy beverages, and tetra-packed drinks, while the present study embedded more groups of sweetened beverages. The most frequently consumed SSB among adolescents in this study was malted drink, which is consistent with that claimed by the MyBreakfast study [34]. Malted drink is manufactured by mixing malt with other cereal and legume flour with or without whole milk or milk powder and/or cocoa powder, in which malted drink consumers were found to have a significantly higher intake of carbohydrates and micronutrients, when compared to non-consumers [34]. Nonetheless, one should be concerned about the sugar content in malted drink. 

The consumption of SSBs among school-aged adolescents in this study seemed to decrease with age. A systematic review performed by Winpenny et al. [35] suggested that the intake of added sugar decreased by age due to the increasing sensitivity in physiological mechanisms towards sucrose, hence the reduction in preference for a sweet taste. On the contrary, several studies reported a positive correlation between age and consumption of SSBs [20,36]. 

This study found that higher physical activity was linked with higher SSB consumption. This result is in agreement with other studies conducted in Texas [15] and Spain [37] among school-aged adolescents. This association may reflect the belief held by adolescents on the benefits of isotonic beverages or sport drinks that aid in water and electrolyte recovery, apart from promoting optimal fitness during or after performing physical activity [38]. 

A higher screen time was related to a higher consumption of SSBs. Similar findings were reported by Wang et al. [39], in which the study found that consuming carbonated drinks at or more than three times was positively linked with high screen time among school-aged adolescents in Zhejiang Province, China. Likewise, another study found that watching television (2.72 times) and other screen times (smartphones, tablets, computers, and/or playing video games (1.98 times)) for five or more hours daily exerted higher tendency to be exposed to higher consumption of SSBs among American high school students [40]. Such an unhealthy association was believed to be influenced by a high exposure to food and beverage advertisements during screen time [18]. At the same time, they paid less attention to what they ate during screen time, which could eventually lead to overconsumption [18].

Poor sleep quality increased the consumption of SSBs among school-aged adolescents in this study. Several past studies reported similar findings [4,18,41]. Several hypotheses have been proposed to explain this association [41]. First, children who slept more had less time to eat. Second, more sleep time reduced screen time and directly reduced their exposure to food and beverages advertisements on televisions and internets. Third, it might be due to the neurohormonal changes that led to reduced sleep time but increased appetite and calorie intake. This particular association could also be due to the intention of short sleepers to consume caffeinated SSBs in order to increase their alertness and to stave off their sleepiness; that is, the excessive consumption of caffeine reduced sleep time [42].

Unhealthy home food availability increased the consumption of SSBs. This finding is in line with other studies [14,19,20,42]. The availability of SSBs at home increased accessibility and eased consumption among adolescents. Parents might influence SSB intake among adolescents as they are the main purchaser in a household [13]. Parents serve as role models and facilitators in impacting children’s diets [14]. A high consumption of SSBs among adolescents could be due to lack of parental rules at home that limit the consumption of SSBs [42]. Nevertheless, parenting practices in the present study was not associated with a higher consumption of SSBs. Perhaps parents were less likely to implement restrictive parenting practices because the children tend to break rules and therefore they seemed ineffective, hence parents allowing their children to consume one to two SSBs on a daily basis [42]. Lack of knowledge pertaining to the negative consequences of SSBs and lack of motivation or parenting skills might function as the promoting factors to a high consumption of SSBs [42]. 

Contrary to other studies [6,21,36], an insignificant association was discovered between BAZ and SSB consumption among adolescents in this study, which is in agreement with other studies [4,43]. The insignificant findings are attributed to the possibility of overweight and obese adolescents who under-reported their SSB intake or a strategy for them to reduce their SSB intake in light of reducing weight [4]. Several confounding factors, such as dieting and body image perception that might influence body weight status, could play an important role in their association with SSB intake, but the variables were omitted from the present study. Furthermore, emerging evidence reveals that frequent SSB consumption is associated with abdominal adiposity, specifically abnormal fat accumulation in visceral adipose tissue [44]. Therefore, abdominal adiposity should be measured in the future in addition of measuring BAZ. More studies are required to confirm the findings.

Several limitations were noted in this study. First, due to the cross-sectional study design, the temporal correlation between risk factors and SSB consumption could not be established. Prospective cohort studies are warranted to support and to verify the findings among school-aged adolescents. Second, this study used a self-reported questionnaire to gather information, which poses a high risk of under- or over-reporting. For instance, the participants had the tendency to under- or overestimate their SSB intake as the outputs relied heavily on their memory to recall the frequency and amount of consumption. The variances in findings of SSB intake may be due to the different definition, measurement, and classification used, primarily because of limited standardized list of SSBs. The other limitation of this study is that the results cannot be generalized to all Malaysian school-aged adolescents because this study only involved adolescents from Gombak, Selangor. Despite these limitations, this study explored the associations of lifestyle factors (physical activity level, screen-viewing behavior, sleep quality, and frequency of eating a meal or snack at fast food restaurant), socio-environmental factors (home food availability, peer social influence, and parenting practice), and body weight status with consumption of SSBs among adolescents in Malaysia, which has not been previously reported. In addition, this study evaluated SSB intake by a continuous measure by incorporating the mean consumption in mL/day. The use of a continuous measure for SSB intake, instead of a dichotomous measure, is preferable to minimize the probability of loss of information [14].

## 5. Conclusions

This study suggests that the daily consumption of SSBs is high among school-aged adolescents. The findings of this study also highlight the unhealthy lifestyle behaviors among school-aged adolescents, such as low physical activity level, high screen time, and poor sleep quality. These unhealthy lifestyles were found to be associated with a high consumption of SSBs. Given the possible negative health outcomes of high SSB intake, it is crucial to ensure efforts in reducing SSB intake among school-aged adolescents. This study points to the need for adolescents to practice a healthy lifestyle, with the aim of reducing SSB intake. Therefore, future interventions may consider incorporating these components in promoting healthy eating behaviors among adolescents. Lifestyle modification is recommended to achieve optimal health and to control the consumption of SSBs among adolescents.

## Figures and Tables

**Table 1:** Characteristics of the participants (*n* = 421).

Characteristic	*n* (%)	Mean ± SD
Sex		
Male	176 (41.8)	
Female	245 (58.2)	
Age (years)		13.30 ± 1.28
12	148 (35.2)	
13	136 (32.4)	
14	14 (3.1)	
15	109 (26.0)	
16	14 (3.3)	
Ethnicity		
Malay	412 (97.9)	
Chinese	2 (0.5)	
Indian	2 (0.5)	
Others	5 (1.2)	
Daily pocket money (MYR)		5.29 ± 2.44
Father’s education level (*n* = 356)		
No formal education	10 (2.8)	
Primary education	6 (1.7)	
Secondary education	272 (76.4)	
Tertiary education	68 (19.1)	
Mother’s education level (*n* = 358)		
No formal education	12 (3.4)	
Primary education	8 (2.2)	
Secondary education	263 (73.4)	
Tertiary education	75 (21.0)	
Monthly household income (*n* = 348)		
<MYR2300.00	105 (30.2)	
MYR2300.00–5599.99	145 (41.7)	
≥MYR5600.00	98 (28.2)	
Physical activity total score		2.13 ± 0.51
Low	280 (68.1)	
Moderate	128 (31.1)	
High	3 (0.7)	
Screen-viewing behavior (hours/day)		
Watch TV/VCD/DVD/music video		
Weekday		2.23 ± 1.49
Weekend		3.66 ± 2.00
Total		2.54 ± 1.39
Play smartphone/computer/video game		
Weekday		1.05 ± 1.25
Weekend		1.81 ± 1.77
Total		1.19 ± 1.15
Total screen time		3.67 ± 1.87
Sleep quality		5.05 ± 2.33
Poor	140 (35.9)	
Good	250 (64.1)	
Frequency of eating a meal or snack at fast food restaurant in the past 7 days		1.57 ± 1.31
None	88 (21.0)	
1 day	155 (36.9)	
2 days	64 (15.2)	
3 days	94 (22.4)	
>3 days	19 (4.5)	
Home food availability		
Healthy home food availability		13.31 ± 3.31
Unhealthy home food availability		8.82 ± 2.72
Fruit and vegetable availability		13.35 ± 3.40
Peers social influence		5.38 ± 2.19
Parenting practice		26.99 ± 5.30
BMI-for-age z-scores		0.52 ± 1.58
Severe thinness	5 (1.2)	
Thinness	18 (4.3)	
Normal	238 (56.7)	
Overweight	76 (18.1)	
Obesity	66 (15.7)	
Severe obesity	17 (4.0)	

Notes. SD = Standard Deviation; MYR = Malaysian Ringgit (USD 1 = MYR 4.14 as on 3 May 2019).

**Table 2:** Total amount of sugar-sweetened beverage (SSB) intake in a day (mL/day).

Types of Beverages	Mean ± SD	Median	Interquartile Range
Fruit juice drink/Fruit drink	97.45 ± 135.01	71.43	0.00–142.86
Regular carbonated flavored drink	70.68 ± 119.28	35.71	0.00–71.43
Isotonic drink	66.49 ± 111.98	35.71	0.00–71.43
Soft drink base/Concentrate	41.60 ± 92.90	0.00	0.00–35.71
Syrup/Cordial (e.g., rose syrup, lychee cordial, grape cordial)	85.71 ± 153.78	35.71	0.00–107.14
Botanical beverages mix (e.g., Chrysanthemum tea, winter melon tea)	67.82 ± 127.00	0.00	0.00–71.43
Soya bean drink/Soya bean milk	74.32 ± 162.50	0.00	0.00–92.86
Cow’s milk	175.78 ± 251.77	92.86	0.00–225.00
Cultured milk/Yogurt drink	120.62 ± 150.66	71.43	0.00–178.57
Tea (e.g., green tea, black tea, red tea)	165.46 ± 233.61	71.43	0.00–250.00
Instant coffee	55.93 ± 123.85	0.00	0.00–46.43
Malted drink	192.05 ± 230.17	107.14	35.71–250.00
Total SSB (without Cow’s milk)	1038.15 ± 725.55	900.00	464.29–1469.64

**Table 3:** Frequency of sugar-sweetened beverage (SSB) intake in a week.

Types of Beverages	*n* (%)							
	On How Many Days a Week Do You Usually Drink Beverages Below?							
	0 day	1 day	2 days	3 days	4 days	5 days	6 days	7 days
Fruit juice drink/Fruit drink	132 (31.4)	106 (25.2)	97 (23.0)	47 (11.2)	17 (4.0)	9 (2.1)	3 (0.7)	10 (2.4)
Regular carbonated flavored drink	192 (45.6)	125 (29.7)	56 (13.3)	25 (5.9)	12 (2.9)	5 (1.2)	2 (0.5)	4 (1.0)
Isotonic drink	195 (46.3)	111 (26.4)	60 (14.3)	31 (7.4)	8 (1.9)	6 (1.4)	1 (0.2)	9 (2.1)
Soft drink base/Concentrate	193 (45.8)	111 (26.4)	61 (14.5)	33 (7.8)	7 (1.7)	5 (1.2)	4 (1.0)	7 (1.7)
Syrup/Cordial (e.g., rose syrup, lychee cordial, grape cordial)	171 (40.6)	106 (25.2)	59 (14.0)	36 (8.6)	15 (3.6)	11 (2.6)	5 (1.2)	18 (4.3)
Botanical beverages mix (e.g., Chrysanthemum tea, winter melon tea)	218 (51.8)	93 (22.1)	39 (9.3)	30 (7.1)	17 (4.0)	8 (1.9)	4 (1.0)	12 (2.9)
Soya bean drink/Soya bean milk	202 (48.0)	97 (23.0)	50 (11.9)	34 (8.1)	14 (3.3)	9 (2.1)	8 (1.9)	7 (1.7)
Cow’s milk	100 (23.8)	85 (20.2)	56 (13.3)	64 (15.2)	31 (7.4)	25 (5.9)	10 (2.4)	50 (11.9)
Cultured milk/Yogurt drink	112 (26.6)	99 (23.5)	57 (13.5)	50 (11.9)	43 (10.2)	24 (5.7)	7 (1.7)	29 (6.9)
Tea (e.g., green tea, black tea, red tea)	116 (27.6)	83 (19.7)	55 (13.1)	50 (11.9)	30 (7.1)	22 (5.2)	20 (4.8)	45 (10.7)
Instant coffee	247 (58.7)	85 (20.2)	28 (6.7)	23 (5.5)	15 (3.6)	6 (1.4)	3 (0.7)	14 (3.3)
Malted drink	71 (16.8)	76 (18.1)	68 (16.2)	59 (14.0)	37 (8.8)	30 (7.1)	18 (4.3)	62 (14.7)

**Table 4:** Factors associated with sugar-sweetened beverages among adolescents.

Characteristics	Simple Linear Regression					Multiple Linear Regression				
	B	Beta	95% CI		*p*-Value	B	Beta	95% CI		*p*-Value
			Lower Bound	Upper Bound				Lower Bound	Upper Bound	
Age	−101.624	−0.178	−155.555	−47.692	<0.001	−108.230	−0.204	−165.904	−50.556	<0.001
Sex	−248.690	−0.169	−387.743	−109.637	<0.001					
Daily pocket money	38.957	0.130	9.357	68.558	0.010					
Physical activity	214.163	0.150	77.125	351.201	0.002	171.581	0.125	24.751	318.411	0.022
Screen time	66.060	0.173	26.779	105.341	0.001	56.013	0.147	15.185	96.842	0.007
Sleep quality	60.913	0.196	30.469	91.357	<0.001	68.441	0.228	35.565	101.318	<0.001
Frequency of eating at fast food restaurants	98.774	0.179	46.439	151.109	<0.001					
Healthy home food availability	−0.459	−0.002	−21.839	20.922	0.966					
Unhealthy home food availability	42.890	0.160	17.358	68.422	0.001	31.372	0.118	2.713	60.031	0.032
Fruit and vegetable availability	1.488	0.007	−19.330	22.306	0.888					
Peers social influence	−29.323	−0.088	−61.403	2.757	0.073					
Parenting practice	−2.701	−0.020	−16.026	10.624	0.690					
BMI-for-age z-scores	−43.379	−0.094	−87.350	0.592	0.053					

Notes. B = Unstandardized coefficients; Beta = Standardized coefficients. Variables with a *p* < 0.25 in the simple linear regression model were included in the stepwise multiple linear regression analysis. Multiple linear regression model: R = 0.387, R^2^ = 0.149, Adjusted R^2^ = 0.135, F = 10.222, *p* < 0.001; associations are significant at *p* < 0.05.
